# Supplementary material for: Factors influencing sepsis associated thrombocytopenia (SAT): A multicenter retrospective cohort study
Source: PLoS One. 2025 Feb 10;20(2):e0318887. doi: 10.1371/journal.pone.0318887 (PMC11809783; doi:10.1371/journal.pone.0318887)
Supplement: Attachment — Submitted filename: PONE-D-24-48544.pdf [file pone.0318887.s001.pdf]

PLOS ONE

Factors influencing sepsis associated thrombocytopenia (SAT): a multicenter retrospective cohort study

--Manuscript Draft--

**Manuscript Number:**	PONE-D-24-48544
**Article Type:**	Research Article
**Full Title:**	Factors influencing sepsis associated thrombocytopenia (SAT): a multicenter retrospective cohort study
**Short Title:**	Factors influencing SAT
**Corresponding Author:**	Xiang Zhou<br>Peking Union Medical College Hospital<br>Beijing, Beijing CHINA
**Keywords:**	sepsis associated thrombocytopenia; Malignancies; norepinephrine equivalents; total bilirubin; PaO2 / FiO2
**Abstract:**	Introduction: Sepsis associated thrombocytopenia (SAT) is a common complication in the intensive care unit (ICU). There is currently a lack of coverage of factors influencing SAT, so we designed this study to investigate factors influencing SAT.<br>Methods:<br>Patients with sepsis (2984 in PUMCH database, 13165 in eICU database, 11101 in MIMIC-IV database) were enrolled. Factors included in this study included the basic information, comorbidities, and organ functions. Basic information included gender, age, and body mass index (BMI). BMI ≥30kg/m^2 was defined as obesity. Comorbidities included diabetes, chronic obstructive pulmonary disease (COPD), hematologic malignancies, and other malignancies. Organ functions included NE equivalents, PaO2 / FiO2, total bilirubin (TBIL), Glasgow Coma Scale (GCS) scores, and creatinine.<br>Main results: In terms of basic information, age and BMI were inversely correlated with the incidence of SAT (p-value 0.175 and 0.049 [PUMCH], p-value 0.000 and 0.000 [EICU], p-value 0.000 and 0.000 [MIMIC-IV]). In terms of comorbidities, hematologic malignancies and other malignancies were positively correlated with the incidence of SAT (p-value 0.000 and 0.000 [PUMCH], p-value 0.000 and 0.000 [EICU], p-value 0.000 and 0.020 [MIMIC-IV]) except other malignancies was inversely correlated with the incidence of SAT in PUMCH database. In terms of organ functions, NE equivalents, TBIL and creatinine were positively correlated with the incidence of SAT (p-value 0.000, 0.000 and 0.011 [PUMCH], p-value 0.028, 0.000 and 0.013 [EICU], p-value 0.028, 0.000 and 0.027 [MIMIC-IV]) while GCS scores was inversely correlated with the incidence of SAT (p-value 0.029 [PUMCH], p-value 0.005 [EICU]). The relationship between PaO2 / FiO2 and SAT was uncertain. PaO2 / FiO2 was inversely correlated with the incidence of SAT in PUMCH database (0.021 [PUMCH]), while it was positively correlated with the incidence of SAT (p-value 0.000 [MIMIC-IV]). PaO2 / FiO2 and SAT was not related (p-value 0.111 [EICU]). TBIL, hematologic malignancies, PaO2 / FiO2 and NE equivalents ranked in the top five significant variables in all three datasets.<br>Conclusions: Hematologic malignancies and other malignancies were positively correlated with the incidence of SAT. NE equivalents, TBIL and creatinine were positively correlated with the incidence of SAT. TBIL, hematologic malignancies, PaO2 / FiO2 and NE equivalents ranked in the top significant variables in factors influencing SAT.
**Order of Authors:**	Lu Wang, MD
	Jieqing Chen
	Xiang Zhou
**Additional Information:**	
**Question**	**Response**
**Financial Disclosure**	Yes

Powered by Editorial Manager® and ProduXion Manager® from Aries Systems Corporation

Enter a financial disclosure statement that describes the sources of funding for the work included in this submission. Review the submission guidelines for detailed requirements. View published research articles from *PLOS ONE* for specific examples.<br><br>This statement is required for submission and **will appear in the published article** if the submission is accepted. Please make sure it is accurate.<br><br>**Funded studies**<br>Enter a statement with the following details:<br>• Initials of the authors who received each award<br>• Grant numbers awarded to each author<br>• The full name of each funder<br>• URL of each funder website<br>• Did the sponsors or funders play any role in the study design, data collection and analysis, decision to publish, or preparation of the manuscript?<br><br>Did you receive funding for this work?	
Please add funding details.<br>as follow-up to "**Financial Disclosure**<br><br>Enter a financial disclosure statement that describes the sources of funding for the work included in this submission. Review the submission guidelines for detailed requirements. View published research articles from *PLOS ONE* for specific examples.<br><br>This statement is required for submission and **will appear in the published article** if the submission is accepted. Please make sure it is accurate.	Xiang Zhou, MD reports financial support was provided by Ministry of Science and Technology of the People's Republic of China (National Key R&D Program of China, No. 2020YFC0861000). Xiang Zhou, MD reports financial support was provided by China Medical Board of United States (China Medical Board, No. CMB 20-381). Lu Wang reports financial support was provided by National Natural Science Foundation of China (National Natural Science Foundation of China, No. 81801901). Xiang Zhou, MD reports financial support was provided by Chinese Academy of Medical Sciences & Peking Union Medical College (National High Level Hospital Clinical Research Funding, 2022-PUMCH-B-115). If there are other authors, they declare that they have no known competing financial interests or personal relationships that could have appeared to influence the work reported in this paper. The funders had no role in study design, data collection and analysis, decision to publish, or preparation of the manuscript.

Powered by Editorial Manager® and ProduXion Manager® from Aries Systems Corporation

**Funded studies**<br>Enter a statement with the following details:<br>• Initials of the authors who received each award<br>• Grant numbers awarded to each author<br>• The full name of each funder<br>• URL of each funder website<br>• Did the sponsors or funders play any role in the study design, data collection and analysis, decision to publish, or preparation of the manuscript?<br><br>Did you receive funding for this work?"	
Please select the country of your main research funder (please select carefully as in some cases this is used in fee calculation).<br>as follow-up to "**Financial Disclosure**<br><br>Enter a financial disclosure statement that describes the sources of funding for the work included in this submission. Review the submission guidelines for detailed requirements. View published research articles from *PLOS ONE* for specific examples.<br><br>This statement is required for submission and **will appear in the published article** if the submission is accepted. Please make sure it is accurate.<br><br>**Funded studies**<br>Enter a statement with the following details:<br>• Initials of the authors who received each award<br>• Grant numbers awarded to each author<br>• The full name of each funder<br>• URL of each funder website<br>• Did the sponsors or funders play any role in the study design, data collection and analysis, decision to publish, or preparation of the manuscript?<br><br>Did you receive funding for this work?"	CHINA - CN
**Competing Interests**	The authors have declared that no competing interests exist.

Use the instructions below to enter a competing interest statement for this submission. On behalf of all authors, disclose any competing interests that could be perceived to bias this work—acknowledging all financial support and any other relevant financial or non-financial competing interests.<br><br>This statement is required for submission and **will appear in the published article** if the submission is accepted. Please make sure it is accurate and that any funding sources listed in your Funding Information later in the submission form are also declared in your Financial Disclosure statement.<br><br>View published research articles from *PLOS ONE* for specific examples.<br><br>**NO authors have competing interests**<br><br>Enter: *The authors have declared that no competing interests exist.*<br><br>**Authors with competing interests**<br><br>Enter competing interest details beginning with this statement:<br><br>*I have read the journal's policy and the authors of this manuscript have the following competing interests: [insert competing interests here]*<br><br>* typeset	
**Ethics Statement**<br><br>Enter an ethics statement for this submission. This statement is required if the study involved:<br><br>• Human participants<br>• Human specimens or tissue<br>• Vertebrate animals or cephalopods<br>• Vertebrate embryos or tissues	The current study was reported in accordance with the Strengthening the Reporting of Observational Studies in Epidemiology Guidelines. This study was conducted in accordance with the Declaration of Helsinki (as revised in 2013). The trial protocol was approved by the Central Institutional Review Board at Peking Union Medical College Hospital (NO. I-24PJ1400), and individual consent for this analysis was waived. There was no identifying or protected health information included in the analyzed dataset.

• Field research<br><br>Write "N/A" if the submission does not require an ethics statement.<br><br>General guidance is provided below. Consult the submission guidelines for detailed instructions. **Make sure that all information entered here is included in the Methods section of the manuscript.**	

Powered by Editorial Manager® and ProduXion Manager® from Aries Systems Corporation

**Format for specific study types**<br><br>**Human Subject Research (involving human participants and/or tissue)**<br>• Give the name of the institutional review board or ethics committee that approved the study<br>• Include the approval number and/or a statement indicating approval of this research<br>• Indicate the form of consent obtained (written/oral) or the reason that consent was not obtained (e.g. the data were analyzed anonymously)<br><br>**Animal Research (involving vertebrate animals, embryos or tissues)**<br>• Provide the name of the Institutional Animal Care and Use Committee (IACUC) or other relevant ethics board that reviewed the study protocol, and indicate whether they approved this research or granted a formal waiver of ethical approval<br>• Include an approval number if one was obtained<br>• If the study involved *non-human primates*, add *additional details* about animal welfare and steps taken to ameliorate suffering<br>• If anesthesia, euthanasia, or any kind of animal sacrifice is part of the study, include briefly which substances and/or methods were applied<br><br>**Field Research**<br><br>Include the following details if this study involves the collection of plant, animal, or other materials from a natural setting:<br>• Field permit number<br>• Name of the institution or relevant body that granted permission	
**Data Availability**<br><br>Authors are required to make all data underlying the findings described fully available, without restriction, and from the time of publication. PLOS allows rare exceptions to address legal and ethical concerns. See the PLOS Data Policy and FAQ for detailed information.	Yes - all data are fully available without restriction

A Data Availability Statement describing where the data can be found is required at submission. Your answers to this question constitute the Data Availability Statement and **will be published in the article**, if accepted.<br><br>**Important:** Stating 'data available on request from the author' is not sufficient. If your data are only available upon request, select 'No' for the first question and explain your exceptional situation in the text box.<br><br>Do the authors confirm that all data underlying the findings described in their manuscript are fully available without restriction?	
**Describe where the data may be found in full sentences. If you are copying our sample text, replace any instances of XXX with the appropriate details.**<br><br>• If the data are **held or will be held in a public repository**, include URLs, accession numbers or DOIs. If this information will only be available after acceptance, indicate this by ticking the box below. For example: *All XXX files are available from the XXX database (accession number(s) XXX, XXX.*).<br>• If the data are all contained **within the manuscript and/or Supporting Information files**, enter the following: *All relevant data are within the manuscript and its Supporting Information files.*<br>• If neither of these applies but you are able to provide **details of access elsewhere**, with or without limitations, please do so. For example:<br><br>*Data cannot be shared publicly because of [XXX]. Data are available from the XXX Institutional Data Access / Ethics Committee (contact via XXX) for researchers who meet the criteria for access to confidential data.*<br><br>*The data underlying the results presented in the study are available from (include the name of the third party*	All relevant data are within the manuscript.

and contact information or URL).<br>• This text is appropriate if the data are owned by a third party and authors do not have permission to share the data.<br><br>* typeset	
Additional data availability information:	

Manuscript

Click here to access/download;Manuscript;Factors influencing SAT1.docx

Factors influencing sepsis associated thrombocytopenia (SAT): a multicenter retrospective cohort study

Short title: Factors influencing SAT

Lu Wang[1†], MD, Jieqing Chen[2], Xiang Zhou[1,2*], MD, on behalf of China National Critical Care Quality Control Centre Group (China-NCCQC)

* Correspondence: zx_pumc@126.com

†Lu Wang and Jieqing Chen contributed equally to this work.

[1]Department of Critical Care Medicine, State Key Laboratory of Complex Severe and Rare Diseases, Peking Union Medical College Hospital, Peking Union Medical College and Chinese Academy of Medical Sciences, Beijing 100730, China.

[2]Information Center Department/Department of Information Management, Peking Union Medical College Hospital, Peking Union Medical College and Chinese Academy of Medical Sciences, Beijing 100730, China.

Monitored email of China-NCCQC group: china_nccqc@163.com

Abstract

Introduction: Sepsis associated thrombocytopenia (SAT) is a common complication in the intensive care unit (ICU). There is currently a lack of coverage of factors influencing SAT, so we designed this study to investigate factors influencing SAT.

Methods:

Patients with sepsis (2984 in PUMCH database, 13165 in eICU database, 11101 in MIMIC-IV database) were enrolled. Factors included in this study included the basic information, comorbidities, and organ functions. Basic information included gender,

age, and body mass index (BMI). BMI ≥30kg/m^2 was defined as obesity. Comorbidities included diabetes, chronic obstructive pulmonary disease (COPD), hematologic malignancies, and other malignancies. Organ functions included NE equivalents, PaO2 / FiO2, total bilirubin (TBIL), Glasgow Coma Scale (GCS) scores, and creatinine.

Main results: In terms of basic information, age and BMI were inversely correlated with the incidence of SAT (p-value 0.175 and 0.049 [PUMCH], p-value 0.000 and 0.000 [EICU], p-value 0.000 and 0.000 [MIMIC-IV]). In terms of comorbidities, hematologic malignancies and other malignancies were positively correlated with the incidence of SAT (p-value 0.000 and 0.000 [PUMCH], p-value 0.000 and 0.000 [EICU], p-value 0.000 and 0.020 [MIMIC-IV]) except other malignancies was inversely correlated with the incidence of SAT in PUMCH database. In terms of organ functions, NE equivalents, TBIL and creatinine were positively correlated with the incidence of SAT (p-value 0.000, 0.000 and 0.011 [PUMCH], p-value 0.028, 0.000 and 0.013 [EICU], p-value 0.028, 0.000 and 0.027 [MIMIC-IV]) while GCS scores was inversely correlated with the incidence of SAT (p-value 0.029 [PUMCH], p-value 0.005 [EICU]). The relationship between PaO2 / FiO2 and SAT was uncertain. PaO2 / FiO2 was inversely correlated with the incidence of SAT in PUMCH database (0.021 [PUMCH]), while it was positively correlated with the incidence of SAT (p-value 0.000 [MIMIC-IV]). PaO2 / FiO2 and SAT was not related (p-value 0.111 [EICU]). TBIL, hematologic malignancies, PaO2 / FiO2 and NE equivalents ranked in the top five significant variables in all three datasets.

Conclusions: Hematologic malignancies and other malignancies were positively correlated with the incidence of SAT. NE equivalents, TBIL and creatinine were positively correlated with the incidence of SAT. TBIL, hematologic malignancies, PaO2 / FiO2 and NE equivalents ranked in the top significant variables in factors influencing SAT.

Keywor[illegible] sepsis associated thrombocytopenia; malignancies; norepinephrine equivalents; total bilirubin; PaO2 / FiO2

Key messages

Sepsis associated thrombocytopenia (SAT) is a common complication in the intensive care unit (ICU). Hematologic malignancies and other malignancies were positively correlated with the incidence of SAT. Norepinephrine (NE) equivalents, total bilirubin (TBIL) and creatinine were positively correlated with the incidence of SAT. TBIL, hematologic malignancies, PaO2 / FiO2 and NE equivalents ranked in the top significant variables in factors influencing SAT.

Introduction

Sepsis associated thrombocytopenia (SAT) is a common complication in the intensive care unit (ICU) [1]. The excessive inflammatory response in sepsis leads to platelet depletion, accompanied by varying degrees of impaired platelet production and increased destruction, resulting in varying degrees of thrombocytopenia, and the degree of thrombocytopenia is closely related to the mortality of patients [2, 3]. SAT is defined as platelet count $<100 \times 10^9$/L or a relative reduction of $\geq$30% from baseline platelet count. Baseline platelet count is defined as the highest value in the past 7 days prior to

ICU admission [4]. There is currently a lack of coverage of factors influencing SAT, so we designed this study to investigate factors influencing SAT.

Methods

Study Design

Based on the third international consensus definitions for sepsis and septic shock, sepsis is life-threatening organ dysfunction caused by a dysregulated host response to infection. In this survey, patients with sepsis admitted to Peking Union Medical College Hospital (PUMCH), in eICU database, in MIMIC-IV database were enrolled. Exclusion criteria included as follows: patients with thrombocytopenia not caused by sepsis (such as hemopathy), patients without platelet count data, patients age less than 18, patients not first admission, and patients with missing > 5% individual data. The data in PUMCH database were collected from June 8, 2013 to October 12, 2022. MIMIC is the largest open source and free clinical database in the critical care and emergency department, based on intensive care inpatient system of Beth Israel Deaconess Medical Center. MIMIC-IV (version 1.0) is the latest version, which contains data from 2008 to 2019. The eICU database was originally drawn from the eICU telehealth system. This system complemented on-site ICU teams with remote support. This multi-center ICU database, comprised over 200,000 admissions to 335 ICUs from 208 hospitals across the USA in 2014 and 2015. We completed the courses required to use the database and obtained the corresponding certificate. The requirement for individual patient consent was waived because the project did not impact clinical care and all protected health information was anonymized. Eventually, patients with sepsis (2984 in PUMCH

database, 13165 in eICU database, 11101 in MIMIC-IV database) were enrolled. The basic information, comorbidities, and organ functions are shown in **Table 1**. Basic information included gender, age, and body mass index (BMI). BMI ≥30kg/m^2 was defined as obesity. Comorbidities included diabetes, chronic obstructive pulmonary disease (COPD), hematologic malignancies, and other malignancies. Organ functions included norepinephrine (NE) equivalents, PaO2 / FiO2, total bilirubin (TBIL), Glasgow Coma Scale (GCS) scores, and creatinine. NE equivalents = NE + epinephrine + phenylephrine/10 + dopamine/100 + metaraminol/8 + vasopressin*2.5 + angiotensin II*10 (all in mcg/kg/min, except vasopressin in units/min) [5].

Table 1. Patient characteristics.

	PUMCH (n = 2,984)	MIMIC (n = 11,101)	eICU (n = 13,165)
Basic information			
Age (years)	58.41±16.24	66.64±15.93	66.45±16.00
Male (%)	1811 (60.70)	6431 (57.90)	6763 (51.40)
BMI (kg/m^2)	24.03±12.52	28.92±7.75	28.82±8.91
Comorbidities (%)			
COPD (%)	1374 (46.00)	7426 (66.90)	1029 (7.80)
Diabetes (%)	1321 (44.30)	7236 (65.20)	1811 (13.80)
Hematologic malignancies (%)	161 (5.40)	493 (4.40)	281 (2.10)
Other malignancies (%)	763 (25.60)	1867 (16.80)	553 (4.20)
Organ function			

NE equivalents	0.216±0.538	0.047±0.124	0.039±0.176
TBIL (μmol/L)	36.08±58.35	24.62±67.30	15.59±40.90
GCS Scores	5.47±2.94	12.69±3.60	11.77±4.16
Creatinine (μmol/L)	116.96±145.14	145.95±145.51	162.74±158.32
PaO2/FiO2 (mmHg)	281.20±313.03	338.04±118.94	250.54±139.05

eICU = eICU database, MIMIC = MIMIC-IV database, PUMCH = Peking Union Medical College Hospital, BMI = body mass index, COPD = chronic obstructive pulmonary disease, NE = norepinephrine, TBIL = Total bilirubin, GCS = Glasgow Coma Scale. NE equivalents = NE + epinephrine + phenylephrine/10 + dopamine/100 + metaraminol/8 + vasopressin*2.5 + angiotensin II*10 (all in mcg/kg/min, except vasopressin in units/min).

The authors are accountable for all aspects of the work in ensuring that questions related to the accuracy or integrity of any part of the work are appropriately investigated and resolved. The datasets supporting the conclusions of this article are included within the article.

Variables and measurements

Firstly, we studied factors influencing SAT from a point of basic information and comorbidities. Secondly, referring to the S[illegible]FA score, we studied the effects of organ functions on SAT from the cardiovascular system, respiratory system, central nervous system, hepatic system, and renal system. Cardiovascular function is represented by NE equivalents. Respiratory function is represented by PO2 / FiO2. Central nervous function is represented by Glasgow Coma Scale score. Hepatic function is represented by plasma levels of total bilirubin. Renal function is represented by plasma levels of

creatinine. Finally, we investigated the effects of SAT on prognosis in septic shock. Prognosis included ICU stays and ICU mortality.

Ethical considerations

The current study was reported in accordance with the Strengthening the Reporting of Observational Studies in Epidemiology Guidelines. This study was conducted in accordance with the Declaration of Helsinki (as revised in 2013). The trial protocol was approved by the Central Institutional Review Board at Peking Union Medical College Hospital (NO. I-24PJ1400), and individual consent for this analysis was waived. There was no identifying or protected health information included in the analyzed dataset.

Data analysis

Continuous variables are expressed as mean ± SD. To determine the factors related to SAT, multi-variable logistic regression models and artificial neural network model were applied. Specifically, the artificial neural network model chosen for this study is the multilayer perceptron, which is a feed-forward artificial neural network model that maps multiple datasets of inputs to a single dataset of outputs. The risk factors were screened according to literature search, expert consensus and expert experience. Factors focused including the patients' baseline characteristics, factors associated with diagnosis and SOFA. The results were expressed as the odds ratio (OR) with 95% confidence interval. In this study, software IBM Modeler (version 18.0) was used to conduct multi-variable logistic regression models and artificial neural network model. All P values presented were two-sided, with $P < 0.05$ being considered

statistically significant. R software (Version 4.1.0) was used in this study for research charting.

Results

Factors influencing SAT and normalized importance in PUMCH database

In terms of basic information, gender and BMI were inversely correlated with the incidence of SAT (p-value 0.000, 0.049). In terms of comorbidities, COPD and hematologic malignancies were positively correlated with the incidence of SAT (p-value 0.037, 0.000) while other malignancies were inversely correlated with the incidence of SAT (p-value 0.000). In terms of organ functions, NE equivalents, and TBIL were positively correlated with the incidence of SAT (p-value 0.000, 0.000, 0.011) while GCS scores was inversely correlated with the incidence of SAT (p-value 0.029, 0.021) **(Fig. 1A)**.

In **Fig. 1B**, normalized importance of all 12 variables were demonstrated. The top five significant variables were NE equivalents, TBIL, hematologic malignancies, PaO2 / FiO2, and creatinine in PUMCH.

Figure 1. Factors influencing SAT and normalized importance in PUMCH database. SAT = sepsis associated thrombocytopenia, PUMCH = Peking Union Medical College Hospital, BMI = body mass index, COPD = chronic obstructive pulmonary disease, NE = norepinephrine, TBIL = Total bilirubin, GCS = Glasgow Coma Scale. NE equivalents = NE + epinephrine + phenylephrine/10 + dopamine/100 + metaraminol/8 + vasopressin*2.5 + angiotensin II*10 (all in mcg/kg/min, except vasopressin in units/min).

Factors influencing SAT and normalized importance in eICU database

In terms of basic information, age and BMI were inversely correlated with the incidence of SAT (p-value 0.000, 0.000) while gender was positively correlated with the incidence of SAT (p-value 0.004). In terms of comorbidities, hematologic malignancies and other malignancies were positively correlated with the incidence of SAT (p-value 0.000, 0.000) while COPD was inversely correlated with the incidence of SAT (p-value 0.000). In terms of organ functions, NE equivalents, TBIL and creatinine were positively correlated with the incidence of SAT (p-value 0.028, 0.000, 0.013) while GCS scores was inversely correlated with the incidence of SAT (p-value 0.005) **(Fig. 2A)**.

In **Fig. 2B**, normalized importance of all 12 variables were demonstrated. The top five significant variables were TBIL, hematologic malignancies, PaO2 / FiO2, NE equivalents, and other malignancies in EICU.

Figure 2. Factors influencing SAT and normalized importance in eICU database. SAT = sepsis associated thrombocytopenia, BMI = body mass index, COPD = chronic obstructive pulmonary disease, NE = norepinephrine, TBIL = Total bilirubin, GCS = Glasgow Coma Scale. NE equivalents = NE + epinephrine + phenylephrine/10 + dopamine/100 + metaraminol/8 + vasopressin*2.5 + angiotensin II*10 (all in mcg/kg/min, except vasopressin in units/min).

Factors influencing SAT and normalized importance in MIMIC database

In terms of basic information, age and BMI were inversely correlated with the incidence of SAT (p-value 0.000, 0.000). In terms of comorbidities, hematologic

malignancies, other malignancies and diabetes were positively correlated with the incidence of SAT (p-value 0.000, 0.020, 0.001). In terms of organ functions, NE equivalents, TBIL, GCS scores, creatinine and PaO2 / FiO2 were positively correlated with the incidence of SAT (p-value 0.028, 0.000, 0.000, 0.027, 0.000) **(Fig. 3A)**.

In **Fig. 3B**, normalized importance of all 12 variables were demonstrated. The top five significant variables were TBIL, hematologic malignancies, PaO2 / FiO2, creatinine and NE equivalents in MIMIC.

Figure 3. Factors influencing SAT and normalized importance in MIMIC database. SAT = sepsis associated thrombocytopenia, BMI = body mass index, COPD = chronic obstructive pulmonary disease, NE = norepinephrine, TBIL = Total bilirubin, GCS = Glasgow Coma Scale. NE equivalents = NE + epinephrine + phenylephrine/10 + dopamine/100 + metaraminol/8 + vasopressin*2.5 + angiotensin II*10 (all in mcg/kg/min, except vasopressin in units/min).

Discussion

Our investigation found that in terms of basic information, age and BMI were inversely correlated with the incidence of SAT. In terms of comorbidities, hematologic malignancies and other malignancies were positively correlated with the incidence of SAT. In terms of organ functions, NE equivalents, TBIL, and creatinine were positively correlated with the incidence of SAT while GCS scores was inversely correlated with the incidence of SAT. TBIL, hematologic malignancies, PaO2 / FiO2 and NE equivalents ranked in the top five significant variables in all three datasets.

Older patients are one of the fastest-growing subgroups of critically ill patients [6].

In Europe, the number of elderly people aged ≥ 80 is increasing rapidly, and the ICU admission rate of patients in this group is also increasing. These populations currently account for about 10% of all ICU admissions in Europe [7]. Immunodeficiency in older patients often results in a deficit in the adaptive immune response, which may lead to decreased consumption of platelet activation in older patients with sepsis, resulting in decreased SAT [8].

Obesity as a protective factor in patients with sepsis has been supported by a growing body of literature [9-11]. Obesity reduces inflammation, protein catabolism, dyslipidemia, and muscle weakness during sepsis [12]. Obesity prevents sepsis-induced browning of white adipose tissue [13]. Obesity improves cellular immune responses, decreases pro-inflammatory cytokine responses, and improves survival by inducing hyperleptinemia in mouse models of sepsis [14]. Our study showed that obesity reduced the incidence of SAT, which may be a pathway for obesity to be a protective factor in patients with sepsis. Further investigation is needed to determine whether this pathway is related to the pathophysiological processes reported in the above literatures.

With the advancement of follow-up diagnosis and treatment technology, the survival rate of patients with malignancy has been significantly improved [15]. The risk of sepsis is significantly higher in the cancer patient population, particularly in patients with hematologic malignancies, compared with the non-cancer population [16, 17]. Immune dysfunction or neutropenia caused by chemotherapy drugs play an important role [18, 19]. Patients with septic shock with hematologic malignancies have a higher mortality rate [16]. Patients with sepsis who have neutropenia have a mortality above

30% [18]. Our study showed that patients with sepsis with malignancy were more likely to develop SAT, which may provide a possible direction for the treatment of patients with such sepsis.

As the most widely used vasoactive drug in the treatment of sepsis [20, 21], norepinephrine has been shown to regulate immunooxidative metabolism and cellular responses, increase anti-inflammatory effects and attenuate pro-inflammatory effects, in addition to vasopressors [22, 23]. Our study showed that patients with larger NE equivalents were more likely to develop SAT. On the one hand, a large amount of NE equivalents indicates that the patient is more critically ill and has a higher incidence of SAT, and on the other hand, whether high-dose vasoactive drugs themselves cause SAT is a question that needs to be answered by further research.

In the SOFA score, the degree of elevated bilirubin represents the severity of hepatic insufficiency in patients with sepsis [24]. Surprisingly, there was a strong correlation between the elevated TBIL and the incidence of SAT, and whether it was related to TBIL itself or overall liver function needs to be answered by further research in the future.

In the SOFA score, the degree of PaO2 / FiO2 decline represents the severity of respiratory insufficiency in patients with sepsis [24]. To our surprise, those with high PaO2 / FiO2 in the MIMIC database were more likely to develop SAT. This may involve mechanisms of regulation of lung ventilation-perfusion ration. Unlike the systemic circulation, the pulmonary circulation is much more sensitive to hypoxemia, especially intra-alveolar hypoxia than intravascular hypoxia. In poorly ventilated intra-alveolar

hypoxic sites, such as lung consolidation, pulmonary vasoconstriction occurs spontaneously by hypoxic pulmonary vasoconstriction and even leads to platelet depletion and thrombosis, thereby improving lung ventilation/blood flow matching [25-27]. In this case, PaO2 / FiO2 improvement is accompanied by thrombocytopenia.

There are several limitations to this study. First, due to indiscriminate attack of the systemic inflammatory response syndrome, there may be simultaneous increase in damage rather than cross-talk between various organ injuries, which may lead to biased results. However, in current clinical practice, the injury to various organs associated with sepsis-related MODS is often not synchronously aggravated [28], which is further confirmed in this study. The correlation between organ function and SAT varied widely, with TBIL and NE equivalents showing a much stronger correlation than other organs, and we even found that patients with higher PaO2 / FiO2 may be more likely to develop SAT in the MIMIC databases. Second, this was a retrospective cohort study and, therefore, prone to selection bias. Therefore, we choose three independent databases to corroborate each other to reduce this bias. Third, this was an observational study and, therefore, the specific pathophysiology behind the observations needs to be answered by further research.

Conclusion

Age and BMI were inversely correlated with the incidence of SAT. Hematologic malignancies and other malignancies were positively correlated with the incidence of SAT. NE equivalents, TBIL and creatinine were positively correlated with the incidence of SAT. TBIL, hematologic malignancies, PaO2 / FiO2 and NE equivalents ranked in

the top significant variables in factors influencing SAT.

Abbreviations:

Sepsis associated thrombocytopenia = SAT, intensive care unit = ICU, Peking Union Medical College Hospital = PUMCH, body mass index = BMI, chronic obstructive pulmonary disease = COPD, norepinephrine = NE, total bilirubin = TBIL, Glasgow Coma Scale = GCS.

Author Contributions:

Data curation: Lu Wang, Jieqing Chen and Xiang Zhou. Methodology: Xiang Zhou. Project administration: Xiang Zhou. Resources: Lu Wang and Jieqing Chen. Supervision: Xiang Zhou. Validation: Xiang Zhou. Writing: Lu Wang and Xiang Zhou. All authors read and approved the final manuscript.

Availability of data and materials: The datasets supporting the conclusions of this article are included within the article.

Ethics approval and consent to participate: The trial protocol was approved by the Central Institutional Review Board at Peking Union Medical College Hospital (NO. I-24PJ1400) and individual consent for this retrospective analysis was waived.

Consent for publication: All authors have agreed to publish it.

Acknowledgements: The authors would like to thank all participants and staff. The China National Critical Care Quality Control Center Group consists of the following persons: Xue Wang, Xiangdong Guan, Yan Kang, Bin Xiong, Bingyu Qin, Kejian Qian, Chunting Wang, Mingyan Zhao, Xiaochun Ma, Xiangyou Yu, Jiandong Lin, Aijun Pan, Haibo Qiu, Feng Shen, Shusheng Li, Yuhang Ai, Xiaohong Xie, Jing Yan, Weidong Wu, Meili Duan, Linjun Wan, Xiaojun Yang, Jian Liu, Hang Xu, Dongpo Jiang, Lei Xu, Zhuang Chen, Guoying Lin, Zhengping Yang, Zhenjie Hu.

References:

1. Ghimire S, Ravi S, Budhathoki R, Arjyal L, Hamal S, Bista A, Khadka S, Uprety D: Current understanding and future implications of sepsis-induced thrombocytopenia. *Eur J Haematol* 2021, 106(3):301-5. https://doi. 10.1111/ejh.13549.
2. Liu Y, Jin G, Sun J, Wang X, Guo L: Recombinant human thrombopoietin in critically ill patients with sepsis-associated thrombocytopenia: A clinical study. *Int J Infect Dis* 2020, 98:144-9. https://doi. 10.1016/j.ijid.2020.06.045.
3. Gonzalez DA, Kumar R, Asif S, Bali A, Dang AK: Sepsis and Thrombocytopenia: A Nowadays Problem. *Cureus* 2022, 14(5):e25421. https://doi. 10.7759/cureus.25421.
4. Zhou H, Li Z, Liang H, Yan Z: Thrombocytopenia and platelet count recovery in patients with sepsis-3: a retrospective observational study. *Platelets* 2022, 33(4):612-20. https://doi. 10.1080/09537104.2021.1970124.
5. Goradia S, Sardaneh AA, Narayan SW, Penm J, Patanwala AE: Vasopressor dose equivalence: A scoping review and suggested formula. *J Crit Care* 2021, 61:233-40. https://doi. 10.1016/j.jcrc.2020.11.002.
6. Kingren MS, Starr ME, Saito H: Divergent Sepsis Pathophysiology in Older Adults. *Antioxid Redox Signal* 2021, 35(16):1358-75. https://doi. 10.1089/ars.2021.0056.
7. Flaatten H, de Lange DW, Artigas A, Bin D, Moreno R, Christensen S, Joynt GM, Bagshaw SM, Sprung CL, Benoit D *et al*: The status of intensive care medicine research and a future agenda for very old patients in the ICU. *Intensive Care Med* 2017, 43(9):1319-28. https://doi. 10.1007/s00134-017-4718-z.
8. Starr ME, Saito H: Sepsis in old age: review of human and animal studies. *Aging Dis* 2014,

5(2):126-36. https://doi. 10.14336/AD.2014.0500126.

9. Yeo HJ, Kim TH, Jang JH, Jeon K, Oh DK, Park MH, Lim CM, Kim K, Cho WH, Korean Sepsis Alliance I: Obesity Paradox and Functional Outcomes in Sepsis: A Multicenter Prospective Study. *Crit Care Med* 2023, 51(6):742-52. https://doi. 10.1097/CCM.0000000000005801.

10. Ng PY, Eikermann M: The obesity conundrum in sepsis. *BMC Anesthesiol* 2017, 17(1):147. https://doi. 10.1186/s12871-017-0434-z.

11. Kalani C, Venigalla T, Bailey J, Udeani G, Surani S: Sepsis Patients in Critical Care Units with Obesity: Is Obesity Protective? *Cureus* 2020, 12(2):e6929. https://doi. 10.7759/cureus.6929.

12. Vankrunkelsven W, Derde S, Gunst J, Vander Perre S, Declerck E, Pauwels L, Derese I, Van den Berghe G, Langouche L: Obesity attenuates inflammation, protein catabolism, dyslipidaemia, and muscle weakness during sepsis, independent of leptin. *J Cachexia Sarcopenia Muscle* 2022, 13(1):418-33. https://doi. 10.1002/jcsm.12904.

13. Li C, Davis X, Lahni P, Stuck J, Williamson L, Kaplan J: Obesity protects against sepsis-induced and norepinephrine-induced white adipose tissue browning. *Am J Physiol Endocrinol Metab* 2021, 321(3):E433-E42. https://doi. 10.1152/ajpendo.00380.2020.

14. Siegl D, Annecke T, Johnson BL, 3rd, Schlag C, Martignoni A, Huber N, Conzen P, Caldwell CC, Tschop J: Obesity-induced hyperleptinemia improves survival and immune response in a murine model of sepsis. *Anesthesiology* 2014, 121(1):98-114. https://doi. 10.1097/ALN.0000000000000192.

15. Siddiqui SS, Prabu NR, Chaudhari HK, Narkhede AM, Sarode SV, Dhundi U, Divatia JV, Kulkarni AP: Epidemiology, Clinical Characteristics, and Prognostic Factors in Critically Ill

Patients with Hematolymphoid Malignancy. *Indian J Crit Care Med* 2021, 25(1):56-61. https://doi. 10.5005/jp-journals-10071-23469.

16. Manjappachar NK, Cuenca JA, Ramirez CM, Hernandez M, Martin P, Reyes MP, Heatter AJ, Gutierrez C, Rathi N, Sprung CL *et al*: Outcomes and Predictors of 28-Day Mortality in Patients With Hematologic Malignancies and Septic Shock Defined by Sepsis-3 Criteria. *J Natl Compr Canc Netw* 2022, 20(1):45-53. https://doi. 10.6004/jnccn.2021.7046.

17. Amanati A, Sajedianfard S, Khajeh S, Ghasempour S, Mehrangiz S, Nematolahi S, Shahhosein Z: Bloodstream infections in adult patients with malignancy, epidemiology, microbiology, and risk factors associated with mortality and multi-drug resistance. *BMC Infect Dis* 2021, 21(1):636. https://doi. 10.1186/s12879-021-06243-z.

18. Malik IA, Cardenas-Turanzas M, Gaeta S, Borthakur G, Price K, Cortes J, Nates JL: Sepsis and Acute Myeloid Leukemia: A Population-Level Study of Comparative Outcomes of Patients Discharged From Texas Hospitals. *Clin Lymphoma Myeloma Leuk* 2017, 17(12):e27-e32. https://doi. 10.1016/j.clml.2017.07.009.

19. Abdolkarimi B, Amanati A, Molavi Vardanjani H, Jamshidi S, Tabaeian SAP: Antibody waning after immunosuppressive chemotherapy and immunomodulators, re-immunization considerations in pediatric patients with malignancy and chronic immune thrombocytopenic purpura. *BMC Infect Dis* 2022, 22(1):657. https://doi. 10.1186/s12879-022-07647-1.

20. Tung M, Crowley JC: Norepinephrine for Early Shock Control in Sepsis. *Am J Respir Crit Care Med* 2019, 200(9):1192. https://doi. 10.1164/rccm.201905-1083LE.

21. El Beze N, Kimmoun A, Asfar P: Early Use of Norepinephrine for Sepsis: Promising Results That Require Confirmation. *Am J Respir Crit Care Med* 2019, 200(9):1191-2. https://doi.

10.1164/rccm.201905-1007LE.

22. Thoppil J, Mehta P, Bartels B, Sharma D, Farrar JD: Impact of norepinephrine on immunity and oxidative metabolism in sepsis. *Front Immunol* 2023, 14:1271098. https://doi. 10.3389/fimmu.2023.1271098.

23. Stolk RF, van der Pasch E, Naumann F, Schouwstra J, Bressers S, van Herwaarden AE, Gerretsen J, Schambergen R, Ruth MM, van der Hoeven JG *et al*: Norepinephrine Dysregulates the Immune Response and Compromises Host Defense during Sepsis. *Am J Respir Crit Care Med* 2020, 202(6):830-42. https://doi. 10.1164/rccm.202002-0339OC.

24. Singer M, Deutschman CS, Seymour CW, Shankar-Hari M, Annane D, Bauer M, Bellomo R, Bernard GR, Chiche JD, Coopersmith CM *et al*: The Third International Consensus Definitions for Sepsis and Septic Shock (Sepsis-3). *JAMA* 2016, 315(8):801-10. https://doi. 10.1001/jama.2016.0287.

25. Gierhardt M, Pak O, Walmrath D, Seeger W, Grimminger F, Ghofrani HA, Weissmann N, Hecker M, Sommer N: Impairment of hypoxic pulmonary vasoconstriction in acute respiratory distress syndrome. *Eur Respir Rev* 2021, 30(161). https://doi. 10.1183/16000617.0059-2021.

26. Licker M, Hagerman A, Jeleff A, Schorer R, Ellenberger C: The hypoxic pulmonary vasoconstriction: From physiology to clinical application in thoracic surgery. *Saudi J Anaesth* 2021, 15(3):250-63. https://doi. 10.4103/sja.sja_1216_20.

27. Gebauer PH, Turzo M, Lasitschka F, Weigand MA, Busch CJ: Inhibition of ornithine decarboxylase restores hypoxic pulmonary vasoconstriction in endotoxemic mice. *Pulm Circ* 2020, 10(4):2045894020915831. https://doi. 10.1177/2045894020915831.

28. Troia R, Mascalzoni G, Calipa S, Magagnoli I, Dondi F, Giunti M: Multiorgan dysfunction

syndrome in feline sepsis: prevalence and prognostic implication. *J Feline Med Surg* 2019, 21(6):559-65. https://doi. 10.1177/1098612X18792106.

Figure 1

Click here to access/download;Figure;Fig 1.tif

A

	Estimate	95% LCI	95% UCI	P value
Age(y)	0.9970	0.9920	1.0010	0.1750
Gender	0.7310	0.6240	0.8550	0.0000
BMI(Kg/m2)	0.7250	0.5260	0.9990	0.0490
Hematologic Malignancies	3.8430	2.5960	5.6900	0.0000
Other Malignancies	0.6490	0.5390	0.7810	0.0000
COPD	1.1840	1.0110	1.3860	0.0370
Diabetes	1.0400	0.8870	1.2180	0.6310
NE equivalents	2.7530	2.0370	3.7190	0.0000
TBIL(μmol/L)	1.0060	1.0040	1.0080	0.0000
GCS Scores	0.9750	0.9530	0.9970	0.0290
Creatinine(μmol/L)	1.0010	1.0000	1.0020	0.0110
PaO2/FiO2(mmHg)	1.0000	0.9990	1.0000	0.0210

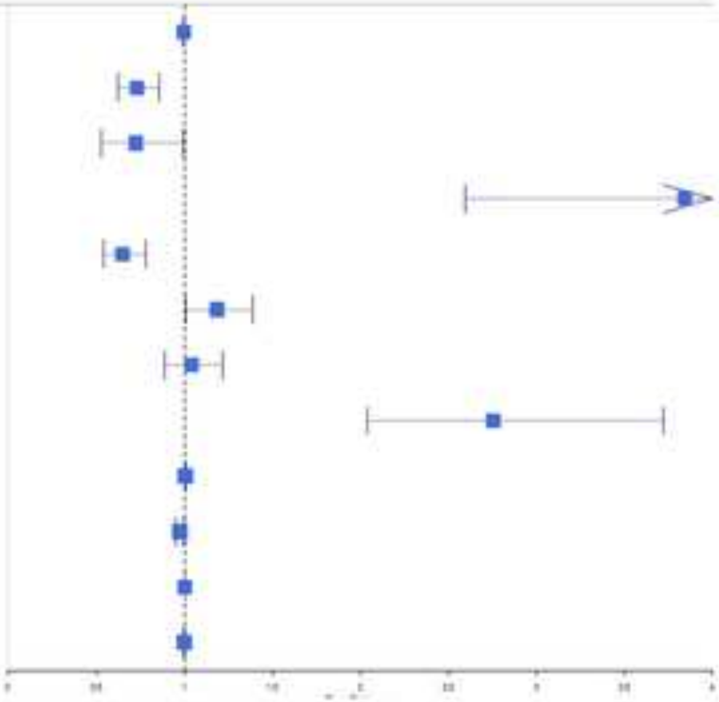

B

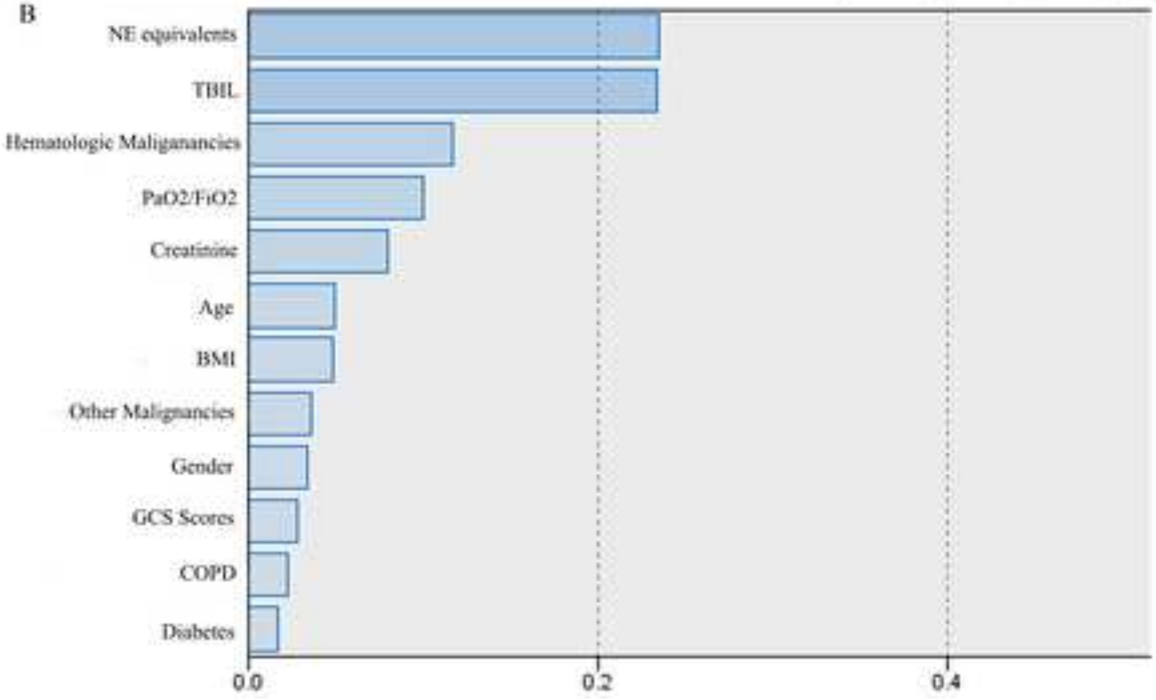

Figure 2

Click here to access/download;Figure;Fig 2.tif

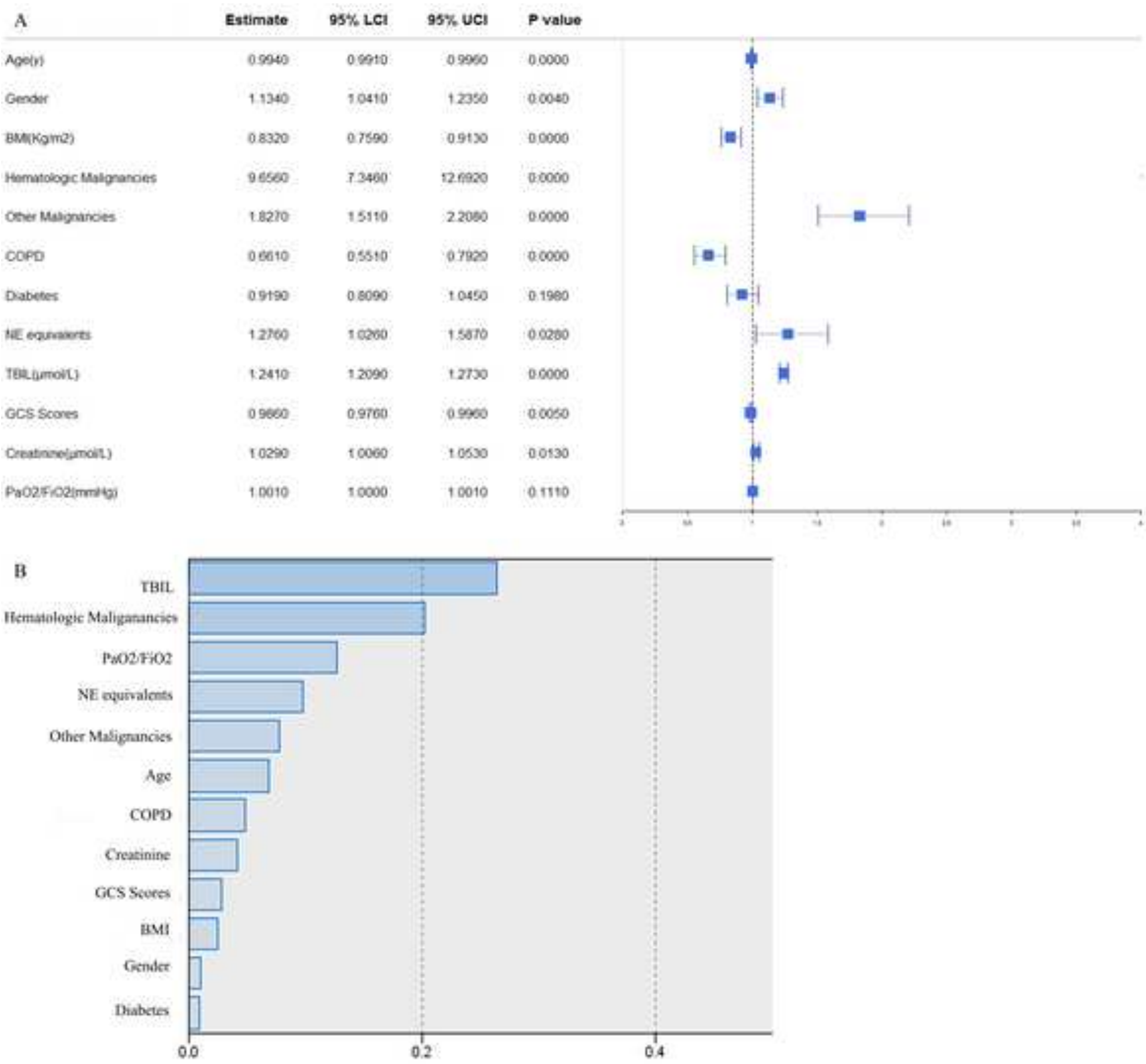

A	Estimate	95% LCI	95% UCI	P value
Age(y)	0.9940	0.9910	0.9960	0.0000
Gender	1.1340	1.0410	1.2350	0.0040
BMI(Kg/m2)	0.8320	0.7590	0.9130	0.0000
Hematologic Malignancies	9.6560	7.3460	12.6920	0.0000
Other Malignancies	1.8270	1.5110	2.2080	0.0000
COPD	0.6610	0.5510	0.7920	0.0000
Diabetes	0.9190	0.8090	1.0450	0.1980
NE equivalents	1.2760	1.0260	1.5870	0.0280
TBIL(μmol/L)	1.2410	1.2090	1.2730	0.0000
GCS Scores	0.9860	0.9760	0.9960	0.0050
Creatinine(μmol/L)	1.0290	1.0060	1.0530	0.0130
PaO2/FiO2(mmHg)	1.0010	1.0000	1.0010	0.1110

Figure 3

Click here to access/download;Figure;Fig 3.tif

A

	Estimate	95% LCI	95% UCI	P value
Age(y)	0.9930	0.9900	0.9960	0.0000
Gender	1.0610	0.9620	1.1690	0.2350
BMI(Kg/m2)	0.7520	0.6680	0.8480	0.0000
Hematologic Malignancies	3.1500	2.6020	3.8120	0.0000
Other Malignancies	1.1610	1.0240	1.3160	0.0200
COPD	0.9910	0.8930	1.1000	0.8680
Diabetes	1.1870	1.0710	1.3160	0.0010
NE equivalents	2.1300	1.4850	3.0560	0.0000
TBIL(μmol/L)	1.1910	1.1700	1.2120	0.0000
GCS Scores	1.0280	1.0140	1.0420	0.0000
Creatinine(μmol/L)	1.0320	1.0040	1.0620	0.0270
PaO2/FiO2(mmHg)	1.0010	1.0010	1.0010	0.0000

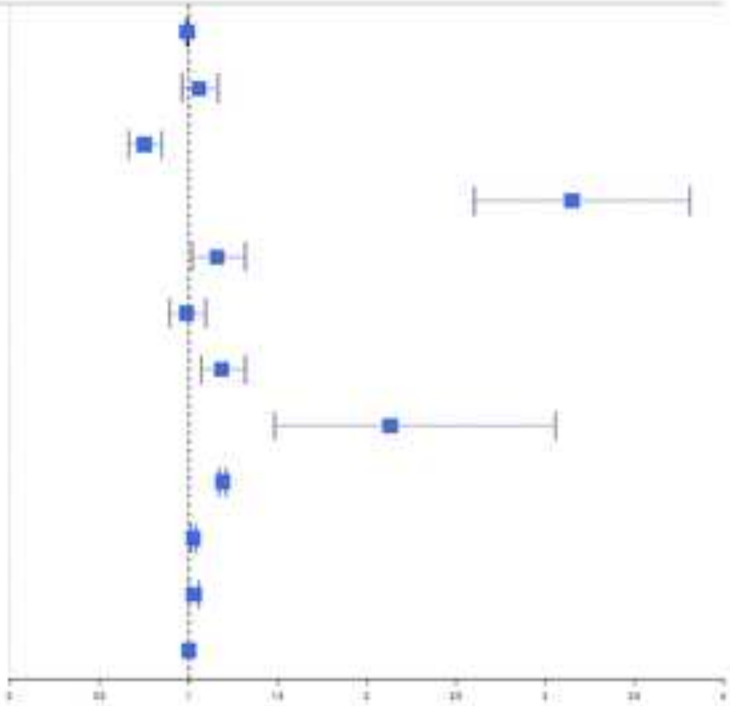

B

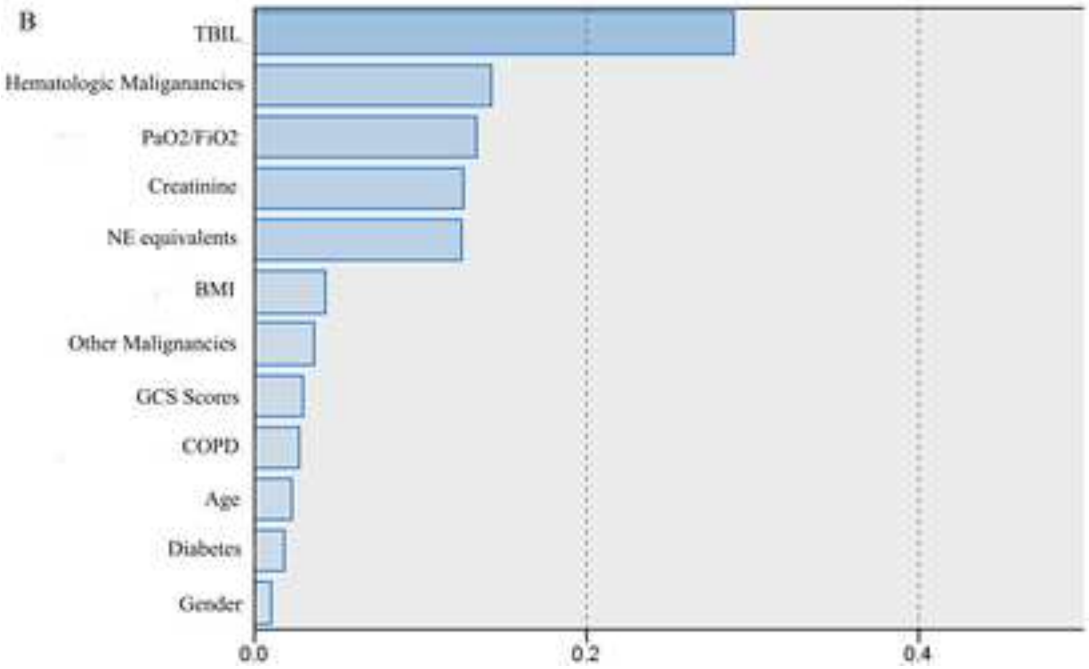

Click here to access/download
Supporting Information
Financial Disclosure.docx
